# Prevalence of trachoma and associated factors in the rural area of the department of Vaupés, Colombia

**DOI:** 10.1371/journal.pone.0229297

**Published:** 2020-05-19

**Authors:** Hollman Alfonso Miller, Clara Beatriz López de Mesa, Sandra Liliana Talero, Mónica Meza Cárdenas, Sandra Patricia Ramírez, José Moreno-Montoya, Alexandra Porras, Julián Trujillo-Trujillo

**Affiliations:** 1 Department of Vaupés, Vector borne and neglected infectious diseases, Mitú, Colombia; 2 Escuela Superior de Oftalmología del Instituto Barraquer de América, Bogotá, Colombia; 3 Subdirection of Communicable Diseases, Ministry of Health and Social Protection, Bogotá, Colombia; 4 Department of Vaupés, San Antonio Hospital of Mitú, Mitú, Colombia; 5 División de Investigaciones Fundación Santa Fe de Bogotá, Bogotá, Colombia; 6 Universidad El Bosque, Bogotá, Colombia; University of Pretoria, SOUTH AFRICA

## Abstract

**Objectives:**

The objectives of the study were to estimate the prevalence of different clinical signs of trachoma and identify possible factors associated with TF.

**Methodology:**

Following the approval of the study protocol by the ethics committee, a cross-sectional study was conducted in Vaupés, a department of the Colombian Amazon, between the years 2012 and 2013 in two districts.

Based on the records obtained from a standardized format for the clinical evaluation of the participants and the factors associated with follicular trachoma, an excel database was built and debugged, which was analyzed using IBM SPSS, Statistics Version 23 and Stata STATA (Version 14, 2015, StataCorp LLC, Texas, USA).

**Results:**

The records of 13,091 individuals was collected from 216 rural indigenous communities, of which 12,080 were examined (92.3%); 7,274 in the Western and 4,806 in the Eastern districts. A prevalence of trachomatous inflammation–follicular (TF) of 21.7% (n = 599; 95% CI 20.2–23.3) in the Western and 24.9% (n = 483; 95% CI 23.1–26.9) in the Eastern district was found in children aged 1 to 9 years. Regarding trachomatous trichiasis (TT), 77 cases were found, of which 14 belonged to the Western district (prevalence 0.3%, CI 95% 0.2–0.5) and 63 to the Eastern district (1.8%, CI 95% 1.4–2.4). Children aged between 1 to 9 years were significantly more likely to have TF when there was the presence of secretions on the face (OR: 3.2; 95% CI: 2.6–3.9).

**Conclusions:**

Trachoma is a public health problem in Vaupés that requires the implementation of the SAFE strategy (S = Surgery, A = Antibiotics, F = Face Washing, E = Environment) in the Eastern and Western districts, for at least 3 consecutive years, in accordance with WHO recommendations.

## Introduction

Trachoma is chronic keratoconjunctivitis, caused by repeated conjunctival infections with serotypes A, B, Ba and C of *Chlamydia trachomatis* [1]. Two phases of the disease are recognized; an acute, self-limiting phase corresponding to the infectious period, and a chronic or advanced phase, generally occurring during adulthood, corresponding to the sequelae of multiple childhood infections. This chronic phase leads to scarring of the tarsal mucosa, entropion, trichiasis, corneal opacity and potential blindness [2].

Trachoma was first identified in the indigenous population of the Colombian Amazon region, in the department of Vaupés [3]. This department is mostly inhabited by indigenous people and characterized by a high cultural and linguistic diversity, poverty, a dispersed population with less than 1 inhabitant per km^2^ [4], poor access to and high operational costs for the provision of healthcare services, in addition to an abundance of water from rivers that cross the whole geography of the jungle. There have been no studies on the prevalence of trachoma conducted in this region.

The department has three urban nuclei: Mitú which is the capital, Carurú and Taraira, and 216 rural communities; it is bordered to the East by the State of Amazonas in the Republic of Brazil, a State and country recognized for its endemicity of trachoma [5], with whom it shares approximately 656 kilometers of border [6]. Historically, there has been a social and economic exchange between the inhabitants of the Colombian and Brazilian Amazon communities and recognized sanitary and hygienic deficiencies, which could explain the presence of trachoma in Vaupés.

The objective of this study was to determine the prevalence of trachoma in the Eastern and Western districts of Vaupés, in order to obtain baseline data and to define the number of years of implementation of the A (antibiotics), F (facial cleanliness) and E (environmental improvement) components of the SAFE strategy before re-survey, according to the WHO recommendations [7,8]. In addition, the identification of associated factors that contribute to the occurrence of TF and which must be addressed by health and other sectors was performed.

## Methods

**Fig 1:**
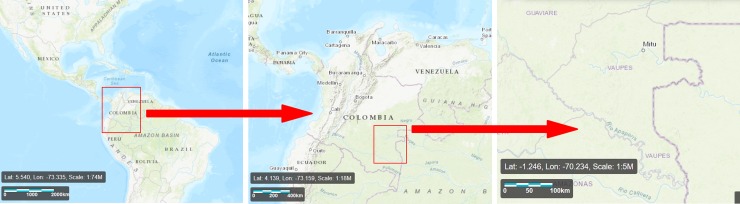
Geographical location of the department of Vaupés, Colombia. Figure was created for this publication using viewer from the USGS LandLook database (https://landsatlook.usgs.gov/viewer.html), January 2020.

To estimate the prevalence of trachoma and its associated factors, a census methodology was performed, in which all known households within the rural communities and single-family or multi-family isolated dwellings of the department of Vaupés were visited. The territory of each ethnic group is distributed to each tribe by ancestry. The indigenous communities and dwellings in the Amazon are located on the banks of the great rivers or their tributaries. The nomadic population has historically been relegated by the other inhabitants to the farthest, most inhospitable and hard-to-reach places in the jungle. However, their temporary settlements are known by health personnel and by members of the work teams that participated as boat drivers, and were therefore also visited for the development of the survey. In each household, all members were included in the census and those who were present and accepted to participate were examined. The rural area in which this study was carried has a surface area of 54,135 km2 [4] and is located in the Northwestern part of the Amazon Region (02°06ʹ North latitude and 01°11ʹ South latitude and between 69°10ʹ and 72°3ʹ West longitude of Greenwich). Fig 1 represents the geographical location of the department in Colombia (Fig 1).

For operational reasons, geographical access and resource availability, the department was divided into two districts, Eastern and Western, which were surveyed in 2012 and 2013 respectively. The urban areas of the municipalities of Mitú and Carurú were excluded, due to the suspicion of a low risk of trachoma transmission.

Prior to the development of the census in the Eastern district, three of the seven graders who executed it, received training in Brazil to perform the external eye examination using 2.5x magnifying glasses and flashlights. In addition, they received instructions in the World Health Organization (WHO) simplified trachoma grading system, by means of which the definition of five clinical signs of trachoma is standardized, two in the sub-acute phase: TF and trachomatous inflammation–intense (TI) and three in the advanced phase of the disease: trachomatous trichiasis (TT), trachomatous scarring (TS) and corneal opacity (CO) [9]. This training lasted one week and included a theoretical and practical component. In 2013, In 2013, the three graders who were trained in Brazil, replicated the training in Mitú to new graders, with four obtaining a kappa of 0.7 or higher and participating in the study. Recorders were trained in the utilization of the different data collection instruments in the census.

Seven work groups were established, each consisting of a grader, a recorder, a boatman and logistical support, who in some areas also served as river guides. Three groups were assigned to the Eastern district and 4 to the Western district. On average, they stayed in each community for 3 days.

All children aged between 1 to 9 years, of all inhabited homes and whose parents or representatives accepted the procedure, were examined in search of sub-acute signs of trachoma. Those over 14 years who gave their consent, were examined for TS, TT and CO. Large-scale treatment was carried out with a single dose of azithromycin (20 mg/kg body weight, maximum 1g), according to the WHO Trachoma Managers' Guide [7]. Education was carried out to inform individuals on the prevention of trachoma, in particular, promoting facial cleanliness, the acceptance of azithromycin and on corrective surgery for trichiasis.

The graders who participated in the fieldwork were supervised during the development of the census at least once by their trainer, in order to verify the correct diagnosis and adherence to the protocol.

For cost-benefit reasons, as an added value and, in accordance with national guidelines for the integration of public health programs and strategies, large-scale co-administration of albendazole with azithromycin (given that, in Vaupés, trachoma and geohelminthiasis overlap in the same populations) was also performed and the collection of data relating to the presence or absence of other diseases were conducted simultaneously with the census, but this analysis is beyond the scope of this study.

A data collection tool was developed (S4 Table) with the variables recorded and analyzed in the Western district including basic demographic aspects such as sex, age, residence, schooling level, ethnic group, settlement pattern (nomadic or sedentary) and clinical diagnosis of trachoma according to the WHO simplified trachoma grading system [9]. In order to guide educational actions and other components of the SAFE strategy, associated factors for trachoma and other overlooked tropical diseases identified in the literature were documented by the recorder during the visit, including sex, age, presence of nasal discharge, dirty hands, place of excreta disposal, distance to the water source, presence of flies on the face, lack of shoes, earthen floor in the household and live with animals; other variables were collected using self-reported data such as: ethnic group and school grade. In the Eastern district, only sociodemographic and clinical variables were collected. TF was the outcome variable to evaluate the associated factors.

The results presented were obtained from the analysis of the nominal databases of both districts and typed in Excel version 8.0. Quality control was performed at 10% of the randomly defined physical records, comparing them with what was entered in the database. In addition, all records with aberrant data identified in the univariate analysis were verified and adjusted from the physical records. All the data were refined, anonymized and analyzed by the Ministry of Health and Social Protection and the *Escuela Superior de Oftalmología del Instituto Barraquer de América*.

In cases where <100% of people were examined because they were absent, a weighting factor was applied to estimate the prevalence of clinical signs of trachoma and the frequency of associated factors. These factors were obtained by dividing 100% (expected value of people to be examined), by the proportion of people in each age group and district actually examined (1 to 9 years and ≥15 years).

The data were processed using STATA (Version 14, 2015, StataCorp LLC, Texas, USA). Chi-square was used to identify associations between categorical variables in the bivariate analysis and Wald test in the multivariate analysis. The Odds Ratio (OR) with its respective 95% confidence interval (95% CI) was used for the raw analysis of the TF association in children from 1 to 9 years of age and the other variables of interest in the Western district; those with biological plausibility or with *p*-values <0.25 were considered as candidates for a logistic regression model that allowed the ORs to be adjusted [10–12]. A value of *p*< 0.05 was considered statistically significant.

The representatives of the departmental indigenous organizations gave their endorsement to the development of the study, whose protocol was approved by the Ethics Committee of the *Escuela Superior de Oftalmología del Instituto Barraquer de América*, Colombia, through certificate number 18–007.

## Results

### Characteristics of the population studied

The census data was compiled from the 216 rural communities of the department of Vaupés; 82 in the Eastern district and 134 in the Western, in which 13,091 people were listed and 12,080 (92.3%) were examined. Differences between people listed and examined is explained by individuals participation in hunting, fishing and agricultural tasks and their subsequent absence during the study visit. Details on the population by age group in each district and in the total rural area of Vaupés are presented in supporting Table 1 (S1 Table).

**Table 1:** Sociodemographic characteristics of the population examined. Colombia, Vaupés. 2012–2013.

Characteristics	Western	Eastern	Total rural area
	Examined (%)	Examined (%)	Examined (%)
**Sex**			
Male	3,745 (51.5)	2,460 (51.2)	6,205 (51.4)
Female	3,529 (48.5)	2,346 (48.8)	5,875 (48.6)
**Total**	**7,274**	**4,806**	**12,080**
**Age**			
Median	22 (IQR 6–40)^a^	21 (IQR 7–39)^a^	21 (IQR 7–40)^a^
1 to 9 years old	2,758 (37.9)	1,769 (36.8)	4,527 (37.5)
15 years and older	4,516 (62.1)	3,037 (63.2)	7,553 (62.5)
**Total**	**7,274**	**4,806**	**12,080**
**Ethnicity**			
Indigenous	7,078 (97.3)	4,746 (98.8)	11,824 (97.9)
Non-indigenous	196 (2.7)	60 (1.2)	256 (2.1)
**Total**	**7,274**	**4,806**	**12,080**
**Settlement pattern (nomadic habits)**			
No	7,165 (98.5)	2,123 (44.2)	9,288 (76.9)
Yes	54 (0.7)	145 (3.0)	199 (1.7)
No information	55 (0.8)	2,538 (52.8)	2,593 (21.4)
**Total**	**7,274**	**4,806**	**12,080**
**School grade**		*	
Without instruction	1,422 (19.5)		
First grade	124 (1.7)		
Second grade	3,210 (44.1)		
Third degree	1,158 (15.9)		
Fourth grade	181 (2.5)		
Fifth grade	27 (0.4)		
Sixth grade	6 (0.08)		
No information	287 (3.9)		
Does not apply^b^	859 (11.8)		
**Total**	**7,274**	**4,806**	**12,080**

^a^ Interquartile range

^b^ This group includes children between 1 and 3 year of age, who have not reached the age to start studies at school

*Information about school grade in the Eastern district was not collected

The distribution by district of the main sociodemographic characteristics of the studied population is presented in Table 1.

We observed that 80% of the communities had less than 100 inhabitants; this information includes *malocas* (isolated indigenous, multi-family housing). Details of the distribution of the communities according to the number of inhabitants in both districts are presented in support Table 2 (S2 Table).

**Table 2:** Distribution of the weighted prevalence of the clinical signs of trachoma according to district. Colombia, Vaupés 2012–2013.

Clinical signs and age of the evaluated	Western district		Eastern district		Total Vaupés				
	n^a^/N^b^ (%)^c^	95% CI ^d^	n^a^/N^b^ (%)^c^	95% CI ^d^	n^a^/N^b^ (%)^c^	95% CI ^d^	OR^e^	95% CI ^f^	*P-value* ^*g*^
TF 1 to 9 years old	599/2,758 (21.7)	20.2–23.3	483/1,939 (24.9)	23.1–26.9	1,083/4,697 (23.0)	21.9–24.3	1.2	1.0–1.4	0.010
TI 1 to 9 years old	45/2,758 (1.6)	1.2–2.2	217/1,939 (11.2)	9.9–12. 7	262/4,697 (5.6)	5.0–6.3	7.6	5.5–10.5	<0.001
TS ≥ 15 yeards old	400/4,750 (8.4)	7.7–9.2	435/3,431 (12.7)	11.6–13.8	835/8,181 (10.2)	9.6–10.9	1.6	1.4–1.8	<0.001
TT ≥ 15 years old	14/4,750 (0.3)	0.2–0.5	63/3,431 (1.8)	1.4–2.4	77/8,181 (0.9)	0.8–1.2	6.5	3.6–11.7	<0.001
CO ≥ 15 years old	2/4,750 (0.04)	0.01–0.2	43/3,431 (1.3)	0.9–1.7	45/8,181 (0.6)	0.4–0.7	28.6	7.2–114.2	<0.001

^a^ Number of people with the clinical sign

^b^ Number of people examined

^c^ Prevalence as a percentage

^d^ 95% Confidence Interval of prevalence

^e^ Odds Ratio to compare the prevalence of each trachoma clinical sign in the Eastern and Western districts

^f^ 95% Confidence Interval of the Odds Ratio

^g^ Test for differences in prevalence between districts. Statistical significance, Chi square test.

The population was predominantly indigenous in both districts with more than 97% classified in this group. Forty different tribes were identified, 32 of them originating from the department of Vaupés, the remaining 8 from other departments of Colombia and Brazil, but recently established in Vaupés. Of the indigenous population examined, the most representative tribe was Cubea (33.8%; n = 3,122), followed by Desana (6.8%; n = 632), Tucana (6.1%; n = 560), Guanana (5.6%; n = 517), and Siriana (5.2%; n = 478).

No statistically significant differences were observed in the median age (Kruskal-Wallis *p = 0*.*637*) or sex (Chi^2^ *p = 0*.*748*) of both districts. However, statistically significant differences were observed by ethnic composition (Chi^2^ *p<0*.*0001*) and nomadic habits of the population (Chi^2^ *p<0*.*0001*).

With respect to education, we found that the proportion of men who were in school for at least one year was higher than that of women, 86.2% and 76.2 respectively; a difference which was statistically significant (p <0.0001).

The census allowed the identification of 145 nomadic Indigens in the Eastern district and 54 in the Western district, belonging to three tribes, as follows: Cacua, (54 people in the Eastern district and 3 in the Western district); jupdá-makú (89 people in the Eastern district and 15 in the Western district) and Yujup-makú (2 people in the Eastern district and 36 people in the Western district).

### Prevalence of clinical signs of trachoma

To compensate for the children aged 1 to 9 years and those over 15 years of age, who were not examined, a prevalence was weighted using the quotient between 100, divided by the proportion of people examined in each of the mentioned groups. Table 2 presents the weighted data, while support Table 3 illustrates this data with and without weighting (S3 Table).

**Table 3:** Risk factors and protectors for TF in children from 1 to 9 years of age, Western district. Colombia, Vaupés, 2012–2013.

Variables	TF positives n (%)^a^	Crude OR^b^	95% CI^c^	*p-value*^d^	Adjusted OR^e^	95% CI^f^	*P-value*^g^
**Sex**	** **	** **	** **	** **	** **	** **	** **
Male	318 (53.1)	1			1		
Female	281 (46.9)	0.8	0. 7–1.0	**0.019**	**0.8**	**0.7–1.0**	**0.019**
**Age**				** **			
1 to 4 years old	255 (42.6)	1.9	1.6–2.3	**<0.001**	**1.6**	**1.3–2.0**	**<0.001**
5 to 9 years old	344 (57.4)	1		** **	1		
**Ethnic group**				** **			
Non-indigenous	16 (2.7)	1		** **	1		
Indigenous	583 (97.3)	1.4	0.8–2.4	**0.135**	1.0	0.6–1.9	0.897
**Settlement pattern**							
Sedentary	588 (98.1)	1					
Nomadic	4 (0.7)	1.2	0.4–3.8	0.745			
No information	7 (1.2)						
**Water location**							
≤ 50 m from the house	465 (77.6)	1					
50 to 200 m outside house	78 (13.0)	0.9	0. 7–1.2	0.349			
≥ 200 m from the house	49 (8.2)	1.2	0.9–1.7	0.257			
No information	7 (1.2)						
**Final disposal stools**							
With latrine	116 (19.4)	1			0.9	0.7–1.2	0.356
In open field >200 m	237 (39.6)	1.2	0.9–1.5	**0.196**	1.2	0.9–1.6	0.136
In open field between 50 and 200 m	235 (39.2)	1.5	1.2–1.9	**0.002**	0.9	0.7–1.26	0.356
No information	11 (1.8)						
**Facial cleanliness and other associated factors**							
No	222 (37.1)	1		** **	1	** **	** **
Secretions on the face (yes)	321 (53.6)	3.5	2.9–4.3	**<0.001**	**3.2**	**2.6–3.9**	**<0.001**
No information	56 (9.3)			** **			
No	219 (36.6)	1		** **	** **	** **	** **
Visibly dirty hands (yes)	336 (56.1)	2.0	1.6–2.4	**<0.001**	0.8	0.6–1.0	0.057
No information	44 (7.3)				** **	** **	** **
No	298 (49.7)	1		** **			
Flies on the face (yes)	229 (38.2)	2.4	1.9–2.9	**<0.001**	1.1	0. 8–1.5	0.619
No information	72 (12.1)						
**Others factors**							
Earthen floor in the household No	433 (72.3)	1					
(yes)	155 (25.9)	0.9	0.7–1.1	**0.177**	1.1	0.9–1.	0.481
No information	11 (1.8)						
Lack of shoes No	8 (1.3)	1					
(yes)	559 (93.2)	1.7	0.8–3.7	**0.150**	1.4	0.7–3.1	0.379
No information	33 (5.5)						
Living with animals No	411 (68.6)	1					
(yes)	187 (31.2)	1.1	0.9–1.4	**0.165**	1.1	0.8–1.4	0.567
No information	1 (0.2)						

^b^ Unadjusted Odds Ratio (from bivariate analysis)

^c^ 95% confidence interval of the Crude Odds Ratio

^d^ Statistical significance X2 of the bivariate analysis

^e^ Adjusted Odds Ratio from the multivariate analysis

^f^ 95% confidence interval of the adjusted Odds Ratio

^g^ Statistical significance Wald Test

Analysis of the results showed statistically significant differences in the prevalence of different clinical signs of trachoma in both districts (Table 2).

### Trachomatous inflammation–follicular

The number of children aged 1 to 9 years with TF was a combined 1,083 from both districts, of whom, 98.4% (n = 1,066) were indigenous. Of the 40 indigenous ethnicities identified, 65% (n = 26) reported at least one case of TF. It was observed that the Cubeos contributed the majority of cases of this clinical sign (22.8%; 95% CI: 21.5–24.1), whilst the remaining cases were distributed heterogeneously among the other 25 indigenous ethnic groups, none of which contributed to more than 6% of cases.

A greater frequency of TF was observed in children between 1 and 4 years old of both sexes, representing 58.1% of all TF cases. There was also a general downward trend in cases from 2 years of age (S1 Fig).

Of the 216 communities visited, children from 1 to 9 years old were identified in 213 of them and screening for sub-acute signs of trachoma was performed. In both districts, 70.4% (n = 150/213) of the communities had prevalence of TF equal to or greater than 5%, 67.2% (n = 88/131) and 75.6% (n = 62/82) in the Western and Eastern districts, respectively. Likewise, the prevalence of TF was greater than or equal to 50% in 20.6% (n = 27/131) and 17.1% (n = 14/82) of the communities of the Western and Eastern districts, respectively. The total prevalence of TF in the department of Vaupés was 23.0%.

Regarding the simultaneous presence of TF and TI, 0.2% prevalence was observed in the Western district (n = 6) and 7.3% (n = 129) in the Eastern.

### Analysis of the factors associated with trachomatous inflammation–follicular

The adjusted OR demonstrated that the associated factors, presence of secretions on the face (nasal or ocular discharge) in children aged 1 to 9 years (OR:3.2; CI 95%:2.6–3.9), children in the age group 1 to 4 years versus children aged between 5 and 9 years (OR: 1.6; CI 95%: 1.3–2.0) and males (OR: 1.25; CI: 1–1.4) were significantly related to the presence of TF (Table 3).

Finally, disposal of excreta in open fields between 50 and 200 m from the house, having visibly dirty hands and the presence of flies on the face was associated with a greater probability of having TF in the bivariate analysis, but not in the multivariate. Table 3 shows the risk and protective factors associated with TF, obtained from the weighted prevalence values of TF for the Western district.

#### Trachomatous inflammation–intense

The prevalence of TI in children aged 1 to 9 assessed in the Western and Eastern districts was 1.6% and 11.2%, respectively, a statistically significant difference *p*<0.001 (Table 2). A greater frequency of TI was found in 2 and 3 year-old children, in whom 41% of cases were identified (n = 108/262). In all cases, TI was also more frequent in the Eastern district (82.8%) and slightly more frequent in the female sex (52.1%). Indigenous ethnic groups accounted for 52.5% of TI cases and was most frequent among the indigenous Itano (n = 21), Cubeos (n = 18), Bará (n = 16), and Tatuya (n = 16). The remaining cases were distributed heterogeneously among the other tribes. For 106 indigenous children no information was obtained about their tribe.

### Trachomatous trichiasis

Individuals aged ≥15 years were screened for TT in the 216 communities surveyed; 77 cases were found, of which 14 (17.8%) were in the Western and 63 (82.2%) in the Eastern districts, representing a prevalence of 0.3% and 1.8% respectively, a statistically significant difference (*p*<0.001). TT was more common in women (66.4%) (n = 51; OR 2.3; 95% CI 1.4–3.6) (S2 Fig), a statistically significant difference (*p*<0.001).

The median age at which TT was observed in the Western and Eastern districts was 56 years (IQR 48–69) and 63 years (IQR: 53–72), respectively. In the Western district, the median age of TT for men was 64 years (IQR: 56–70), compared with 62 years (IQR 49–72) for women. A similar trend was observed in the Eastern district where the median age of TT for men was 58 years (IQR: 48–69) compared with 56 years (IQR: 38–70) for women. The differences in the median age for men and women were not statistically significant.

**Fig 2:**
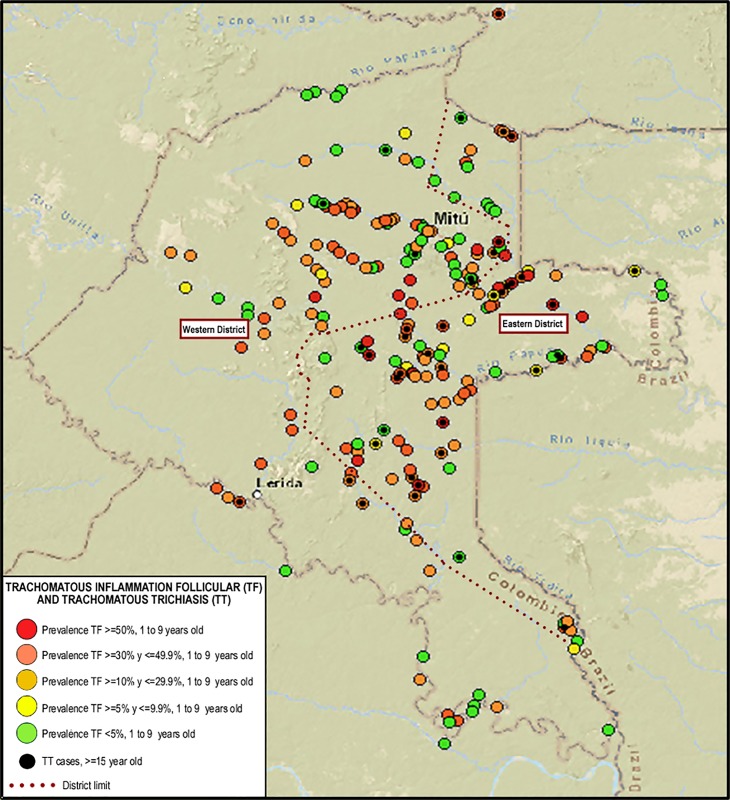
Geographic distribution of TF according to prevalence range by community and cases of TT. Fig 2 was created for this publication using shapefiles from the USGS LandLook database (http://landsatlook.usgs.gov/), December 2018 and QGIS 2.18.14.

The geographical distribution of TT cases in individuals ≥15 years, overlapping over the same communities in which the prevalence of TF data was mapped (indicated by black dots in Fig 2).

The indigenous population contributed to 100% of TT cases (n = 77). It was observed that in the Western district there were TT cases in 25.6% of indigenous ethnic groups (n = 10/39), with a higher frequency in the Bará, Siriana and Tatuya, with two cases each. In the Eastern district, 43.3% of indigenous ethnic groups presented with TT (n = 13/30) and in 27 of the cases, their ethnic affiliation or tribe could not be identified. Desanos were the most affected with five cases.

Of the 14 cases of TT found in the Western district, 6 were women without any degree of education. Of the other eight cases, individuals only reached the second year of basic primary education, 5 of whom were women. In the Eastern district 62.5% of TT cases were in women (n = 35/56). Information about the educational level of these individuals was not known.

Other clinical signs of trachoma such as TS and CO were evaluated and their prevalence is presented in Table 2. A wide confidence interval was observed for the OR of corneal opacity, most likely associated with the low number of people identified with this clinical sign in the Western district.

## Discussion

The results of this survey place both districts of the department of Vaupés on the endemic list for trachoma, with a rural prevalence of TF in children aged 1 to 9 years of 21.7% in the Western district and 24.9% in the Eastern district. Consequently, both districts are eligible for the implementation of the SAFE strategy, including the large-scale drug administration of azithromycin for at least three years, according to WHO guidelines [7]. The prevalence of TF is similar to those estimated by Sarah Polack et al in the document, “Mapping the global distribution of trachoma” published in the WHO Bulletin for tropical humid areas (17%) [13], and reported among the Yanomami Indians of the Brazilian Amazon (24.9%) [14], and greater than the most recently reported prevalence in school children in an urban community in the State of Amazonas, Brazil, close to Vaupés (9.0%) [5]. Another study published in Brazil nineteen years before this study has demonstrated the endemic situation of trachoma in *Alto Río Negro*, the border between Brazil and Colombia in the year 1999. However, the design and sample size are not comparable with our population-based study and there are no recent studies in the same area [15].

The prevalence of TT was 0.3% and 1.8% in the Western and Eastern districts, respectively. Both districts exceeded the threshold defined by the WHO to consider it a public health problem "a prevalence of trachomatous trichiasis unknown to the health system of >0.2% in people aged ≥15 years (approximately 1 case per 1000 total population)” [16], and therefore requiring the implementation of the SAFE strategy. However, although these prevalences are close to this cut-off point, the high dispersion of the population, the difficult geographical access of patients to health services and the high costs for displacement are seen as the main difficulties in eliminating trachoma.

The main factor associated with the increased prevalence of TF, was the presence of nasal and eye discharge in children. Although the measurement of facial cleanliness is subjective and difficult to standardize [17], inadequate facial cleanliness and risk practices among indigenous children and caregivers in Vaupés endemic communities is evident. This situation is also influenced by the lack of resources to buy soap or forgetting to use it in cleaning routines, as reported by Kristen Aiemjoy et al. in Ethiopia [18]. Considering that the non-use of soap can increase the probability of having active trachoma [19] and that there are difficulties for its permanent supply in the middle of the jungle [20], it represents a challenge for the implementation of the F component of the SAFE strategy. There also appears to be a lack of perception concerning the risk that nasal and ocular discharge represents in the transmission of trachoma in the indigenous population of Vaupés, which is to say, the presence of these secretions visibly on the face of children seems to be culturally accepted.

Given the diversity of ethnic groups in the department of Vaupés described in our study, it is important that the SAFE strategy is adapted both socio-culturally and technically, including novel approaches to achieve acceptance of surgical procedures to correct trichiasis and improve intercultural education. Similarly, the topical administration of azithromycin drops (Azydrop®) for three days (in the morning and at night), to improve treatment compliance in children under 1 year, instead of 6 weeks use of ointment of oxytetracycline as recommended by the WHO [7], should also be adopted, amongst other strategies to ensure adherence to treatment. This is of particular importance as unpublished reports from the staff that supervised or distributed azithromycin in the Colombian Amazon reported the lack of adherence to tetracycline ointment in children under 6 months of age, evidenced by the finding of the unused drug during subsequent visits. They also mentioned the low acceptance of azithromycin in suspension in children 6 months to 1 year of age, unlike Azydrop®, which were mostly accepted and with which, some evidence suggests the same effectiveness as the oral medication [21].

In contrast to the situation reported in African countries endemic for trachoma and in Australian aborigines, Vaupés has an abundance of water sources, with 93.3% of its rural communities located less than 200 meters from large and small rivers, with variations in summer not limiting access. Water availability is also complemented by high annual rainfall [22] and the routine of its indigenous inhabitants in collecting rainwater in containers supplied by the local government. Analyzing the presence of TF and its relationship to distance from home to the water source for facial cleanliness, found that there was no statistically significant association. This finding and the high proportion of children with dirty faces, advocates the need to educate the population about the link between trachoma and nasal and ocular discharges, and stress the importance of water in hygiene routines, within the framework of component F of SAFE.

In the multivariate analysis of this study, no statistically significant differences were observed in the prevalence of TF in children who have a toilet seat in their homes and those who deposit fecal matter between 50 and 200 meters, or more than 200 meters from the home. This finding is consistent with other studies in which no association was found [18,23–25], although contradicts that observed by Meredith E. Stock et al [21]. However, the interpretation of this result in our study should be performed with care due to the use of latrines, which were not investigated in the survey. It was evidenced that many people with latrines in their homes still preferred to deposit their excreta in open fields, far from their homes. This was done, as it was argued that the land was not suitable for the implementation of toilet seats, due to the high water table, and the risk factor that flooding posed, as described by the Pan American Health Organization in its document, “Latrines in flood-prone areas” [26].

We found 38.2% of the children surveyed for TF in Western district had flies on their faces, but this did not correlate with a higher probability of developing TF (AOR 1.1 CI 95% 0.8–1.5; *p =* 0.619). This finding is contradictory to other studies that suggest the role of flies as a mechanical vector of trachoma [27–29]. However, we believe that further studies are needed to clarify the role and vector capacity of these flies, and its function in the transmission of trachoma. The presence of flies has previously been identified in the entomology laboratory of the Vaupés health authority as the Chloropidae family, belonging to the genera Hippelates and Liohippelates [3]. At present, there is little research available concerning this family, and increasing our understanding of the Chloropids bionomy is important given that the limited bibliographical information available suggests that it does not rest, nor reproduces in fecal matter [29]. Therefore, a program that promotes the provision and use of latrines to control trachoma in these areas would potentially be ineffective. This hypothesis is also supported by the absence of evidence of *Musca sorbens* in this Amazon department, which is a species of fly involved as a mechanical vector in endemic countries of Africa [30].

It was discovered that the probability of developing TT was higher in women compared to men (OR 2.3; 95% CI: 1.4–3.6; *p*<0,001) and significantly higher in the Eastern district than in the Western district (OR 6.5; CI 95% 3.6–11.7; *p*<0.001). Our explanation for the differences by gender is attributed to the role assumed by the indigenous woman of the Amazon since childhood, in the care of the youngest children in the household. As documented in the literature [31,32], children carry the highest burden of TF and consequently, can result in an increased risk of transmission from the child to their caregiver. Concerning the difference in TT prevalence between the two districts, an explanation for this is beyond the scope of this study.

During the survey, no sociodemographic or hygiene and sanitation information was collected in the Western district, therefore, the risk and protective factors were only studied in the Eastern district, a limitation of our study, and as in any cross-sectional study, we recognize the possible presence of unassessed confounding factors that could confuse the associations described for TF. We also recognize that a significant proportion of nomadic indigenous people registered in both districts could not be examined, 78% (n = 87/145) in the East and 70% (n = 38/54) in the West. This is due to their hunting and gathering activities when health workers visited their temporary settlements. To address this problem, we performed weighted prevalence calculations for each trachoma clinical sign.

Given the epidemiological link between Vaupés and other departments and districts of the Amazon, with whom the same social health determinants are shared, it is considered relevant to carry out a trachoma diagnosis program in the Amazon basin. This should transcend the political and administrative borders of the region and therefore should include, Venezuela, Brazil and Peru, with whom large border areas are shared, in order to determine the prevalence of trachoma and the magnitude of this health problem.

## Supporting information

S1 Fig Distribution of TF cases in children from 1 to 9 years old, according to age and sex. (TIF)

S2 Fig Distribution of TT cases in people aged 15 and over, by sex. (TIF)

S1 Table Representativeness in the survey of participants, according to age group and district. Vaupés 2012–2013.
(XLSX)

S2 Table Distribution of the communities by number of inhabitants listed by district. (XLSX)

S3 Table Distribution of the weighted prevalence of the clinical signs of trachoma according to district. Colombia, Vaupés 2012–2013.
(XLSX)

S4 Table Data collection tool. (XLSX)
