# Supplementary figures and images for: Prevalence of trachoma and associated factors in the rural area of the department of Vaupés, Colombia
Source: PLoS One. 2020 May 19;15(5):e0229297. doi: 10.1371/journal.pone.0229297 (PMC7237033; doi:10.1371/journal.pone.0229297)

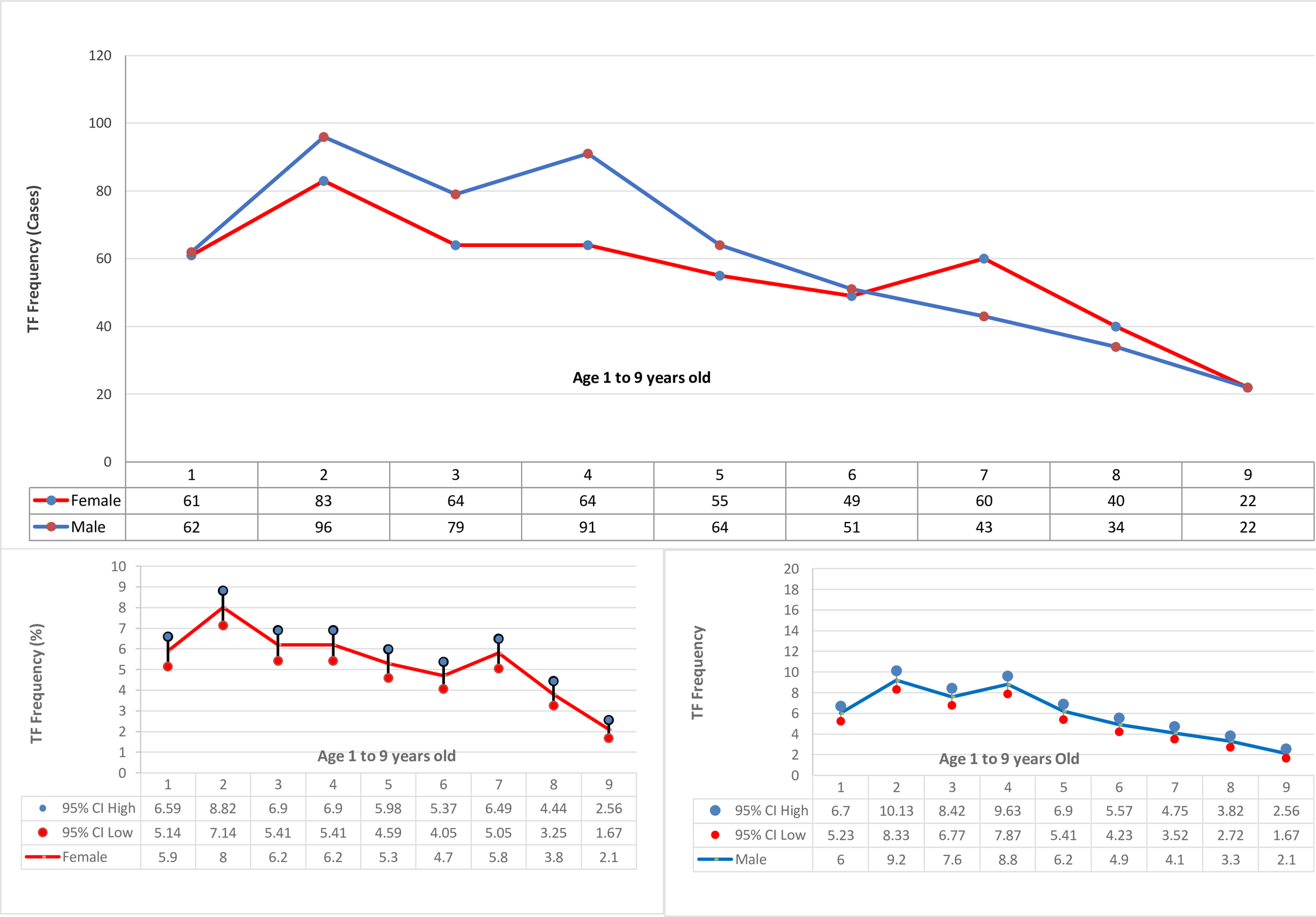

Supplement: S1 Fig — (TIF) [file pone.0229297.s001.tif]

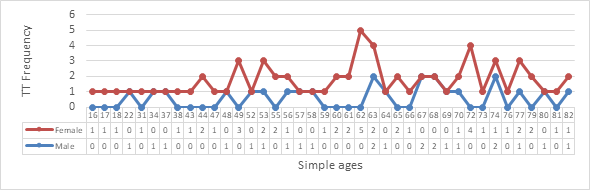

Supplement: S2 Fig — (TIF) [file pone.0229297.s002.tif]
